# Evidence of Early Diabetic Nephropathy in Pediatric Type 1 Diabetes

**DOI:** 10.3389/fendo.2021.669954

**Published:** 2021-04-28

**Authors:** Leena Mamilly, Lucy D. Mastrandrea, Claudia Mosquera Vasquez, Brett Klamer, Mahmoud Kallash, Ahmad Aldughiem

**Affiliations:** ^1^ Section of Pediatric Endocrinology, Nationwide Children’s Hospital, The Ohio State University College of Medicine, Columbus, OH, United States; ^2^ Division of Endocrinology/Diabetes, UBMD Pediatrics and University at Buffalo/Oishei Children’s Hospital of Buffalo, NY, United States; ^3^ Section of Pediatrics, Nationwide Children’s Hospital, The Ohio State University College of Medicine, Columbus, OH, United States; ^4^ Biostatistics Resource at Nationwide Children’s Hospital, Nationwide Children’s Hospital, Columbus, OH, United States; ^5^ Section of Pediatric Nephrology, Nationwide Children’s Hospital, The Ohio State University College of Medicine, Columbus, OH, United States; ^6^ Section of Pediatric Nephrology, Dayton Children’s Hospital, Dayton, OH, United States

**Keywords:** diabetes, nephropathy, biomarkers, blood pressure, glucose variability

## Abstract

**Background:**

Diabetic nephropathy (DN) is one of the most common microvascular complications in type 1 diabetes Mellitus (T1D). Urinary markers of renal damage or oxidative stress may signal early stages of DN. The association of these markers with blood pressure (BP) patterns and glycemic variability (GV) in children is yet to be explored.

**Methods:**

Subjects between the ages of 10 and 21 years with T1D were enrolled. Continuous glucose monitoring (CGM) and ambulatory blood pressure monitoring (ABPM) were performed on each subject. Urine samples were collected and analyzed for albumin, creatinine, neutrophil gelatinase-associated lipocalin (NGAL) and pentosidine.

**Results:**

The study included 21 subjects (62% female) with median age of 16.8 (IQR: 14.5, 18.9). Median HbA1C was 8.4 (IQR: 7.5, 9.3). While microalbuminuria was negative in all but one case (4.8%), urinary NGAL/Cr and pentosidine/Cr ratios were significantly elevated (P<0.001) in diabetic patients despite having normal microalbuminuria, and they correlated significantly with level of microalbumin/Cr (r=0.56 [CI: 0.17, 0.8] and r=0.79 [CI: 0.54, 0.91], respectively). Using ABPM, none had hypertension, however, poor nocturnal systolic BP dipping was found in 48% of cases (95% CI: 28-68%). Urinary NGAL/Cr negatively correlated with nocturnal SBP dipping (r=-0.47, CI: -0.76, -0.03). Urine NGAL/Cr also showed a significant negative correlation with HbA1c measurements, mean blood glucose, and high blood glucose index (r=-0.51 [CI: -0.78, -0.09], r=-0.45 [CI: -0.74, -0.03], and r=-0.51 [CI: -0.77, -0.1], respectively). Median urinary NGAL/Cr and pentosidine/Cr ratios were higher in the high GV group but were not significantly different.

**Discussion:**

This pilot study explores the role of ABPM and urinary markers of tubular health and oxidative stress in early detection of diabetic nephropathy. GV may play a role in the process of this diabetic complication.

## Background

Diabetic nephropathy (DN) is one of the most frequent microvascular complications of diabetes mellitus, affecting 25 to 40% of patients with type 1 diabetes (T1D) (1). It is the single most common cause of end stage renal disease (ESRD) in adults in the Western world (1, 2). Diabetic nephropathy is marked by pathological changes occurring in the renal glomeruli that lead to the development of albuminuria, hypertension, and progressive decline in renal function (3). Hypertension is both a feature of, and a risk factor for the progression of diabetic nephropathy (4). Studies using ambulatory blood pressure monitoring (ABPM) have examined early patterns of blood pressure abnormalities in individuals with diabetes and the link to renal disease progression. Lurbe et al. found that abnormal nocturnal dipping of systolic blood pressure (SBP) was linked to progression of microalbuminuria (5) while Marcovecchio et al. found that daytime diastolic blood pressure was most predictive of future microalbuminuria (6).

Traditionally, measurement of microalbuminuria (urine microalbumin/Cr ratio: 30 to 300 mg/g) has been used in the clinical setting to screen for diabetic nephropathy. However, evidence suggests that renal pathological changes such as nephromegaly and glomerular basement membrane thickening develop soon after diabetes diagnosis and much earlier than microalbuminuria can be detected (7–9). Several serum and urine markers are increased in children with T1D compared to healthy controls (10, 11) signaling the different aspects of pathophysiology involved in this complication. Of interest to our study, urinary neutrophil gelatinase-associated lipocalin (uNGAL) has been studied extensively as a marker of tubular damage in both acute (12) and chronic kidney disease of various etiologies (13–17). Urinary pentosidine is an advanced glycation end product (AGE) that marks oxidative stress caused by tissue exposure to hyperglycemia, and levels of urine pentosidine are elevated in patients with T1D (10). Changes in the levels of these markers in the urine has been documented in patients with T1D. However, correlation with clinical features of diabetic nephropathy such as blood pressure patterns and with glycemic risk factors has not been fully established.

While the role of overall hyperglycemia, as measured by glycated hemoglobin (HbA1c), in the pathogenesis of diabetic kidney disease is well established (18), the role of glycemic variability (GV) is an area of debate. *In-vitro* studies have demonstrated that glycemic excursions are more deleterious to human endothelial cells than sustained hyperglycemia (19). GV has been linked clinically to the development of diabetic retinopathy and neuropathy in both T1 and T2 diabetes (20, 21). Using self-measured blood glucose levels, earlier studies concluded that GV was not associated independently with diabetes complications (22). However, more recent studies using continuous glucose monitoring (CGM) data demonstrated a role for GV in diabetic nephropathy (23). The specific contribution of GV to the disease process in various stages of DN is yet to be elucidated.

The current study was designed to evaluate the use of NGAL and Pentosidine as urinary markers of DN in children with T1D and their relationship with ABPM patterns. Secondarily, we studied the association of glycemic variability measures as well as measures of glycemic control (HbA1c) with the levels of urinary markers of nephropathy.

## Methods

This is a cross-sectional study. Subjects between the ages of 10 and 21 years with T1D for more than 1 year were recruited from the Diabetes and Nephrology pediatric outpatient clinics. Given the known effects of puberty on both glycemic control and microvascular complications, the age range of cases was chosen to avoid prepubertal population. Patients were excluded if they had type 2 or monogenic diabetes, obesity (BMI ≥95th percentile or ≥30 kg/m2 for patient ≥ 18 years of age), secondary hypertension unrelated to diabetes, chronic kidney disease from another etiology, chronic inflammatory diseases, acute infection, or a history of malignancy. Consents and assents were obtained from all participates. Controls (n=10) were enrolled from Nephrology- Urology clinics.

The study entailed a single visit for each case. Weight (kg), height (cm) and manual blood pressure in the seated position were obtained. Pubertal staging was assessed by a pediatric endocrinologist or a pediatric endocrinology fellow. Random urine samples were collected at the time of the visit. ABPM was performed for 24 hours and CGM for 3 to 7 days. Electronic medical records were reviewed for the most recent HbA1c (within 1 month of enrollment) and for lipid panel. Records were also reviewed for a recent serum Creatinine (Cr) value to estimate the renal function; most cases did not have a Cr value within one year of enrollment and thus was not included in the analysis. During the monitoring period, cases continued their usual diabetes care as recommended by their pediatric endocrinologist.

For the controls, they only provided a urine sample and did not get ABPM or CGM.

### Laboratory Analysis

All participants were asked to provide a first morning sample, but most samples were midday samples. After collection, urine samples (subjects N= 21, control N= 10) were centrifuged and the supernatant aliquoted and stored at -80°C for later analysis. Urine albumin was analyzed by ELISA (*ThermoFisher Scientific^®^. Waltham, MA*). For analysis of the urine creatinine, the urine samples were first deproteinized using 10 kD spin columns (*ThermoFisher Scientific^®®^. Waltham, MA*). Enzymatic colorimetric assay was used for analysis of creatinine on the deproteinized sample (*Abcam^®^.Cambridge, UK*). Urine NGAL (Boster Bio Tech Co. Pleasanton, CA) and pentosidine (MyBioSource. San Diego, CA) were analyzed by ELISA. All urine markers were represented as ratios to urine creatinine to adjust for hydration status.

### Analysis of ABPM Data

24 hour ABPM readings were obtained with an automatic oscillometric method device (Spacelab monitor) to all cases. The ABPM monitor was set for measurements at 20 minute intervals during the day and 30 minute intervals during the night. All cases were asked to record their bedtime and wake-up time while wearing the monitor and were not on blood pressure medications. Systolic and diastolic BP during daytime, nighttime and average BP were compared to the reference value based on sex and height (24). Nocturnal blood pressure dipping was defined as the percentage fall of systolic and diastolic measurements during the night compared with the respective measurements during the day. Nocturnal dipping of 10% or above was considered normal whereas dipping of less than 10% was considered abnormal.

### Analysis of CGM Data

CGM data were obtained using a professional monitoring system (Freesyle Libre Pro, Abbott Diabetes Care Inc.). Sensors were placed on the upper arm for all cases and continued for 3-7 days. CGM readings were downloaded using the secure company website (www.libreview.com), and raw data were extracted for analysis. Measures of glycemic variability (10) were calculated using EasyGV software (EasyGV by Nathan R Hill. ^©^University of Oxford 2010+). The primary measure of glycemic variability used in this study was the coefficient of variation (25); the standard cutoff of 36% was used to distinguish between patients with stable (less variability) vs. unstable (more variability) blood glucose levels.

### Statistical Analysis

Descriptive statistics are summarized as median (interquartile range [IQR]) or as frequency (percent). The Wilcoxon rank sum test was used for comparing continuous variables and Fisher’s exact test was used to test for categorical relationship within ABPM groups and GV groups. Spearman’s correlation was used for summarizing the relationship between continuous clinical variables. Scatterplots were used to examine the relationship between logarithmically-transformed urinary markers and their independent variables. Two-tailed *p* values of < 0.05 were considered as statistically significant. Analyses were performed using the R system for statistical computing (R Core Team, 2020), version 3.6.3, and Graphpad Prism 7.

## Results

The study enrolled 21 cases. **Table 1** shows the subject characteristics. More females participated in the study (62%). Three cases (14%) met the ADA recommended glycemic targets of 2019 ADA standards (26). Controls (N= 10) were similar in characteristics of sex, race, and BMI, though tended to be slightly younger at enrollment (**Table 1**). Controls had no significant renal disease (idiopathic microscopic hematuria 6, elevated BP 2, stones 1, joint hypermobility 1). Most cases (81%) used continuous subcutaneous insulin infusion for diabetes management at the time of study enrollment. Eleven cases (52%) had LDL cholesterol levels ≥100 mg/dl; none of the study cases had levels exceeding 130 mg/dl (**Table 2**). Median HbA1C of study cases was 8.4% (IQR: 7.5, 9.3). Retinal exam results were available for 18 (86%) study cases and none had evidence of diabetic retinopathy.

**Table 1:** Baseline demographics of study cases.

Characteristic	cases, N = 21^1^	Controls, N = 10^1^	p-value^2^
**Age**	16.8 (14.5, 18.9)	14.5 (11.6, 15.2)	**0.021**
**Sex**			0.7
Female	13 (62%)	5 (50%)	
Male	8 (38%)	5 (50%)	
**Race**			>0.9
Caucasian	19 (90%)	10 (100%)	
African American	1 (4.8%)	0 (0%)	
Other	1 (4.8%)	0 (0%)	
**Height (cm)**	163.4 (161.2, 171.4)	160.9 (152.8, 170.4)	0.2
**Weight (kg)**	59.7 (54.6, 73.6)	47.2 (37.6, 67.2)	0.12
**BMI (kg/m2)**	22.6 (19.9, 24.9)	19.3 (15.9, 24.4)	0.12

^1^Median (IQR); n (%).

^2^Wilcoxon rank sum test; Fisher’s exact test. BMI, Body mass index.

**Table 2:** Baseline clinical characteristics of study cases.

Characteristic	N = 21^1^
**Tanner stage**	
1	0 (0%)
2	2 (9.5%)
3	1 (4.8%)
4	5 (24%)
5	13 (62%)
**Diabetes duration (years)**	5.0 (3.8, 7.9)
**Total daily insulin dose (U/kg)**	0.8 (0.7, 0.9)
**HbA1c**	8.4 (7.5, 9.3)
**Manual SBP (mm Hg)**	108.0 (104.0, 110.0)
**Manual DBP (mm Hg)**	68.0 (65.0, 70.0)
**LDL-c**	101.5 (85.2, 116.2)
**HDL-c**	54.5 (47.5, 61.0)
**Total cholesterol mg/dl**	178.0 (156.8, 186.2)

^1^n (%); Median (IQR). BP, blood pressure; S, systolic; D, diastolic; SBP, systolic blood pressure; DBP, diastolic blood pressure; LDL- C, low-density lipoprotein cholesterol; HDL- C, high-density lipoprotein cholesterol; U/Kg, unit/Kilogram.

### Blood Pressure

Manual BP measurements were normal for all study cases. ABPM findings are outlined in **Table 3**. Notably, 10 (50%) subjects had abnormal nocturnal dipping of systolic blood pressure but none of the subjects met criteria for hypertension or nocturnal hypertension. No differences in baseline characteristics were found between cases with normal vs. abnormal nocturnal dipping of systolic blood pressure (**Table 4**). Nocturnal dipping in systolic blood pressure showed significant negative correlation with uNGAL/Cr but not with other urinary markers (r=-0.47, CI: -0.76, -0.03; **Table 4**).

**Table 3:** Ambulatory blood pressure monitoring data for the study cases.

Characteristic	N = 21^1^
**Overall SBP (mm Hg)**	107.0 (103.0, 111.2)
**Overall DBP (mm Hg)**	66.0 (62.8, 68.2)
**Daytime SBP (mm Hg)**	109.0 (105.0, 116.0)
**Daytime DBP (mm Hg)**	68.0 (65.0, 73.0)
**Nighttime SBP (mm Hg)**	98.5 (94.8, 103.0)
**Nighttime DBP (mm Hg)**	57.0 (55.0, 59.2)
**SBP dipping (mm Hg)**	9.9 (6.2, 14.4)
**DBP dipping (mm Hg)**	16.6 (12.4, 22.4)
**MAP dipping (mm Hg)**	12.4 (8.4, 16.1)

^1^Median (IQR).

BP, blood pressure; S, systolic; D, diastolic; MAP, mean arterial pressure; SBP, systolic blood pressure; DBP, diastolic blood pressure..

**Table 4:** Baseline characteristics of the case groups with normal nocturnal dipping in systolic blood pressure and abnormal dipping.

Characteristic	Normal dipping, N = 10^1^	Abnormal dipping, N = 10^1^	p-value^2^
**Age**	17.0 (15.0, 18.2)	16.0 (14.6, 18.9)	0.9
**Sex**			0.2
Female	4 (40%)	8 (80%)	
Male	6 (60%)	2 (20%)	
**BMI (kg/m2)**	20.7 (19.5, 25.7)	23.1 (22.4, 24.6)	0.4
**Diabetes duration (years)**	4.7 (3.4, 7.1)	6.0 (4.5, 8.9)	0.4
**Total daily dose (U/kg)**	39.6 (37.1, 48.6)	49.1 (41.4, 52.4)	0.4
**HbA1c**	8.3 (7.5, 9.3)	8.5 (7.7, 9.1)	>0.9
**Manual SBP (mm Hg)**	107.0 (100.0, 119.0)	110.0 (104.0, 110.0)	0.6
**Manual DBP (mm Hg)**	65.0 (64.2, 67.5)	69.0 (68.0, 70.0)	0.043
**LDL-c (mg/dL)**	98.0 (76.0, 118.0)	103.5 (97.0, 115.8)	0.7
**TG (mg/dL)**	86.0 (56.0, 93.0)	87.5 (64.0, 129.0)	0.3
**Overall SBP (mm Hg)**	108.5 (105.2, 110.5)	104.5 (102.2, 112.5)	0.4
**Overall DBP (mm Hg)**	68.0 (64.5, 70.5)	64.0 (62.2, 66.0)	0.2
**Mean blood glucose (mg/dL)**	197.0 (180.4, 230.1)	200.7 (164.8, 266.8)	0.8
**Blood glucose CV**	34.5 (30.2, 40.1)	41.4 (34.5, 50.4)	0.2
**Creatinine (mg/mL)**	0.1 (0.1, 0.1)	0.1 (0.1, 0.1)	0.4
**Urine albumin/Cr**	12.5 (10.5, 21.0)	16.5 (11.2, 20.8)	0.7
**Urine NGAL/Cr**	21.1 (11.3, 78.1)	44.8 (18.1, 172.8)	0.2
**Urine pentosidine/Cr**	88.5 (75.0, 102.8)	113.5 (95.5, 148.2)	0.14
**Systolic BP dipping**	14.5 (12.2, 15.6)	5.7 (0.8, 7.7)	
**Diastolic BP dipping**	21.1 (17.5, 24.4)	13.8 (8.6, 16.2)	0.021

^1^Median (IQR); n (%).

^2^Wilcoxon rank sum test; Fisher’s exact test.

BP, blood pressure; S, systolic; D, diastolic; TG, Triglycerides; CV, coefficient of variation; BMI, Body mass index; U/ Kg, unit/Kilogram; SBP, systolic blood pressure; DBP, diastolic blood pressure; LDL- C, low-density lipoprotein cholesterol; Cr, creatinine; NGAL, Neutrophil gelatinase-associated lipocalin..

### Urinary Biomarkers

**Figure 1:**
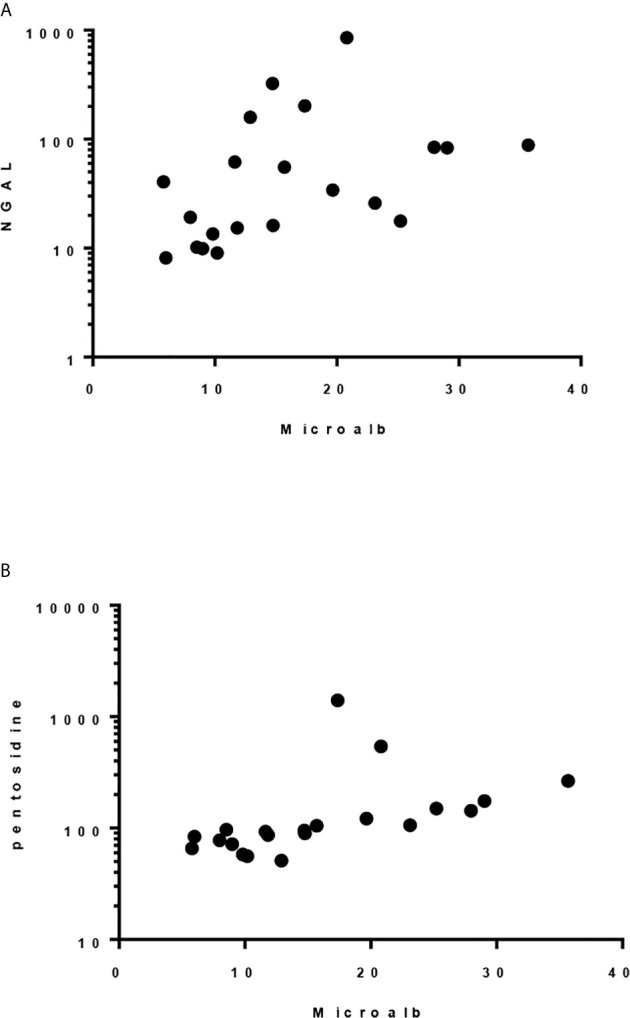
Spearman correlation between urinary microalbumin/Cr and: **(A)** uNGAL/Cr, r = 0.5662, *p* 0.0075 and **(B)** Pentosidine/Cr. r = 0.787, *p < *0.0001.

Although there was only one case had abnormal urine microalbumin/creatinine ratio (36 mcg/g), urine NGAL/Cr and pentosidine/Cr were significantly elevated compared to the controls (P< 0.001, **Table 5**). Significant correlation was found between level of urine microalbumin/Cr and both NGAL/Cr and pentosidine/Cr (r= 0.566, *p* = 0.008 and r= 0.787, *p <*0.001, respectively; **Figure 1**). There was no correlation between urinary biomarkers and duration of DM.

**Table 5:** Urinary markers in diabetes cohort compared to healthy controls.

Characteristic	cases, N = 21^1^	Controls, N = 10^1^	p-value^2^
**Creatinine (mg/mL)**	0.1 (0.1, 0.1)	1.9 (1.9, 2.0)	<0.001
**uNGAL/Cr (ng/mg)**	34.2 (15.4, 84.2)	3.7 (3.2, 6.3)	<0.001
**uAlb/Cr (ug/mg)**	14.7 (9.8, 20.8)	0.7 (0.6, 0.9)	<0.001
**uPentosidine/Cr (pmol/mg)**	95.0 (78.0, 143.0)	13.9 (13.3, 14.8)	<0.001

^1^Median (IQR)

^2^Wilcoxon rank sum test. U, urine; NGAL, Neutrophil gelatinase-associated lipocalin; Alb, albumin; Cr, creatinine.

### Glucose Monitoring

CGM data were available for all study cases. For glycemic variability, we used the coefficient of variation (CV) >36%, a cutoff proposed by (25). Twelve cases (57%) had elevated CV. The study cohort was stratified base on CV, and the main characteristics of the groups are shown in **Table 6**. The lower CV group had higher observed blood glucose index (HBGI) compared with the higher CV group, but we did not find evidence against the null hypothesis (p=0.1). We did find evidence that the lower CV group had higher than the expected values for both HbA1c and mean blood glucose (p=0.028 and p=0.015, respectively). There was no significant correlation between GV measurers and ABPM readings, including nocturnal SBP dipping.

**Table 6:** Baseline characteristics of the case group with high glycemic variability (BG CV≥36%) and group with low variability (BG CV<36%).

Characteristic	CV<36%, N = 9^1^	CV≥36%, N = 12^1^	p-value^2^
**Age**	14.8 (13.6, 17.2)	17.1 (14.8, 19.0)	0.2
**Sex**			0.7
Female	5 (56%)	8 (67%)	
Male	4 (44%)	4 (33%)	
**BMI (kg/m2)**	22.6 (20.2, 26.7)	22.4 (19.7, 24.1)	0.8
**Diabetes duration (years)**	4.4 (3.8, 5.0)	7.2 (4.1, 9.9)	0.2
**Total daily dose (U/kg)**	40.3 (39.0, 52.7)	43.5 (36.2, 50.1)	>0.9
**HbA1c**	9.6 (8.2, 10.5)	8.1 (7.2, 8.7)	**0.028**
**Manual SBP (mm Hg)**	110.0 (104.0, 110.0)	106.0 (103.5, 111.5)	0.7
**Manual DBP (mm Hg)**	68.0 (65.0, 70.0)	68.0 (65.8, 70.0)	>0.9
**LDL-c (mg/dL)**	106.5 (83.2, 118.2)	101.5 (86.8, 108.8)	0.8
**TG (mg/dL)**	98.0 (54.8, 110.2)	86.0 (60.5, 87.2)	0.8
**Overall SBP (mm Hg)**	106.0 (104.0, 109.0)	108.0 (101.5, 113.0)	0.8
**Overall DBP (mm Hg)**	66.0 (64.0, 71.0)	65.0 (61.5, 68.0)	0.2
**Mean blood glucose (mg/dL)**	282.8 (206.4, 349.7)	186.7 (157.2, 200.7)	**0.015**
**Blood Glucose SD**	68.8 (61.9, 96.9)	80.5 (71.7, 93.8)	0.7
**Blood glucose CV**	30.8 (29.0, 33.5)	44.3 (40.3, 53.2)	
**LBGI** ^3^	4.0 (0.4, 6.8)	4.1 (3.2, 10.9)	0.4
**HBGI** ^3^	30.2 (14.3, 45.9)	16.1 (13.6, 18.8)	0.10
**Creatinine (mg/mL)**	0.1 (0.1, 0.2)	0.1 (0.1, 0.1)	0.6
**Urine albumin/Cr**	12.0 (9.0, 25.0)	15.5 (11.5, 20.2)	0.6
**Urine NGAL/Cr**	19.3 (9.9, 83.6)	37.4 (16.0, 116.9)	0.4
**Urine pentosidine/Cr**	84.0 (72.0, 143.0)	101.0 (89.2, 158.0)	0.2

^1^Median (IQR); n (%).

^2^Wilcoxon rank sum test; Fisher’s exact test.

^3^See reference (27).

BP, blood pressure; S, systolic; D, diastolic; LBGI, low blood glucose index; HBGI, high blood glucose index; SD, standard deviation; CV, coefficient of variation; BMI, Body mass index; U/Kg, unit/Kilogram; SBP, systolic blood pressure; DBP, diastolic blood pressure; LDL- C, low-density lipoprotein cholesterol; TG, Triglycerides; NGAL, Neutrophil gelatinase-associated lipocalin; Cr, creatinine..

**Figure 2:**
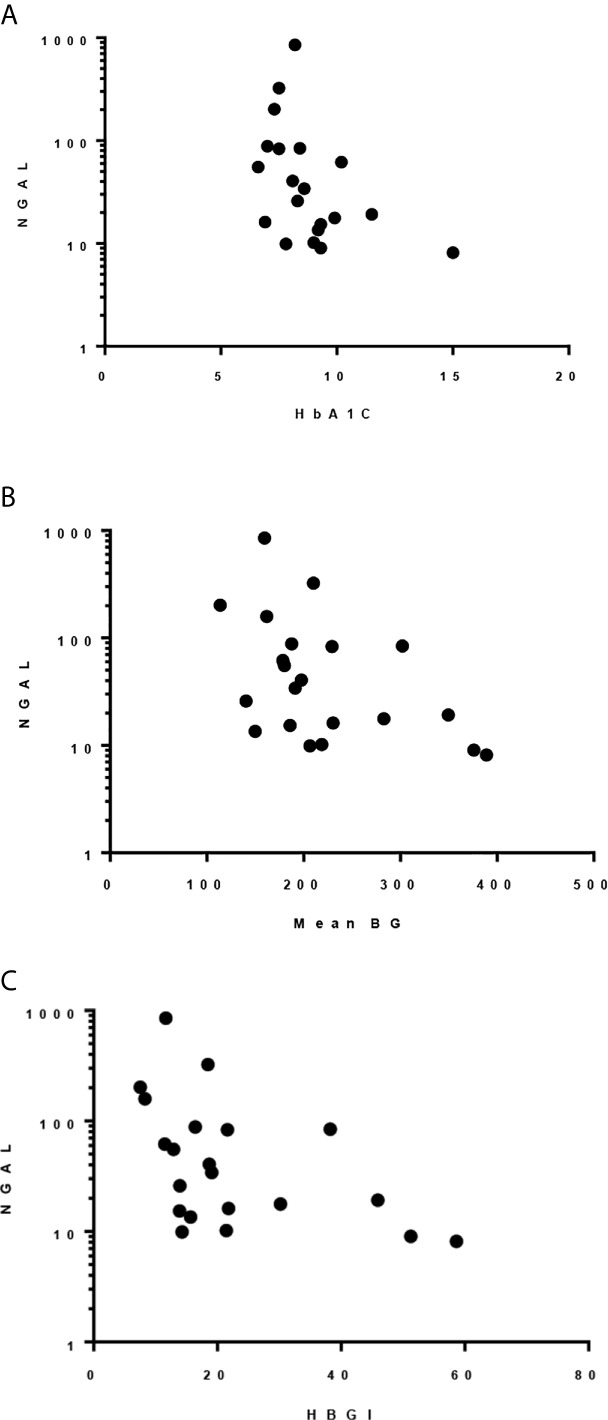
Spearman correlation between uNGAL/Cr and: **(A)** HbA1c; r -0.509, p 0.022. **(B)** Mean BG; r -0.455, p 0.038. **(C)** HBGI; r -0.512, p 0.018.

HbA1c, mean blood glucose, and HBGI correlated negatively with uNGAL/Cr but not with the other urine markers (r=-0.51 [CI: -0.78, -0.09]; r=-0.45 [CI: -0.74, -0.03]; r=-0.51 [CI: -0.77, -0.1], respectively) (**Figure 2**). We also observed higher median uNGAL/Cr and pentosidine/Cr in high GV group; however, the differences did not reach statistical significance (**Table 6**). We did not find a significant relationship between LGBI and urine biomarkers.

## Discussion

Diabetic nephropathy is a multifactorial, insidious disease process and represents a major complication for individuals with T1D. This study in a pediatric T1D cohort aims to explore the characteristics of BP profile and glucose variability and their correlation with urinary markers of glomerular and tubular damage as well as markers of oxidative stress. We found that subjects with T1D have high urinary NGAL/Cr (marker of tubular injury) and pentosidine/Cr (marker of oxidative stress) than controls, when microalbuminuria was negative. We also found a high prevalence of abnormal blood pressure dipping in our cohort, which is consistent with the results of other studies, including normotensive and normoalbuminuric pediatric T1D patients (28, 29). Abnormal BP dipping has been shown to be a predictor for micro- and macro-vascular complications in individuals with diabetes (28, 30, 31). Urinary NGAL has been shown to be of value in the evaluation and progression of both acute and chronic renal damage of different etiologies (12, 13, 15, 32). T1D patients with microalbuminuria have higher urine NGAL and NGAL/Cr levels compared to those with normal urine microalbumin (33, 34). Our study further demonstrates that even with normal urine microalbumin/Cr, NGAL/Cr correlates with albumin excretion. Furthermore, we demonstrate that urine NGAL correlates with loss of nocturnal dipping of SBP, one of the earliest blood pressure profile abnormalities detected for children with T1D. This highlights the potential role of this urine inflammatory biomarker in the early detection of diabetic nephropathpy prior to the microalbuminuric phase.

Oxidative stress is another prominent contributor to the pathophysiology of DN. Urinary pentosidine is a major component of advanced glycation end (AGEs) products resulting from tissue exposure to hyperglycemia causing oxidative stress. It has been shown to be elevated in children with T1D starting at disease onset. Pentosidine levels are higher in children with microalbuminuria compared to those with normal urine albumin excretion (UAE) and correlate with traditional measures of glycemic control (HbA1c) (11, 35). Our results indicate that urinary pentosidine levels correlate with UAE (even within the normal urinary albumin excretion) as well as with mean arterial blood pressure. These findings add to evidence promoting the use of AGEs as early markers of diabetes complications and potential targets for monitoring and intervention in the early stages of disease progression. The small number of subjects in our study is a limiting factor that might have precluded the elucidation of relationships between urinary pentosidine levels and blood pressure measures.

Since its discovery, HbA1c has been used as the primary measure of metabolic control in people with diabetes, representing the mean average glucose over the preceding 2-3 month period. Apart from that “average”, not a lot is known about how the frequency and/or amplitude of day-to-day and hour-to-hour blood glucose fluctuations might affect complications. Glycemic variability is recognized as a potential risk factor for diabetes complications. The increasing availability of glucose data from CGM systems provides the potential to understand the role of blood glucose excursions in relationship to diabetes comorbidities. In addition, recent advances in insulin pump technologies (Hybrid closed-loop systems) target the reduction of such variability and improve overall diabetes control. Interestingly in our cohort, the higher glycemic variability (higher CV) group tended to have lower HbA1c and would be viewed in “better” glycemic control using this traditional measure of metabolic control. This observation makes our finding of a negative correlation between uNGAL and HbA1C, HBGI, and mean blood glucose levels highly provocative and points towards a potential greater contribution of glycemic excursions in the pathophysiology of nephropathy than the exposure to significant but stable hyperglycemia. Further clarification of relationships between measures of GV and markers of DN as well as blood pressure patterns might be possible with a larger cohort.

Our study had limitations, such as the small number of cases and the lack of long term follow up. However, this study was designed as a pilot in order to explore the feasibility of larger, prospective and multicenter studies looking into the development of DN and mitigation of its risk factors.

In summary, diabetic nephropathy is a multifactorial complication that starts early after diagnosis and eventually has a large impact on patient’s quality of life and prognosis. Our study highlights the high prevalence of abnormal BP pattern as well as identifies early urinary biomarkers of renal injury before the development of microalbuminuria. Our results suggest that higher blood glucose values are not necessarily associated with higher markers of tubular injury, raising the question of whether glycemic variability rather than mean blood glucose has a stronger impact on development of DN. Future research is needed to identify the disease process earlier to allow for intensified intervention and new therapeutic targets.

## Data Availability Statement

The original contributions presented in the study are included in the article/supplementary material. Further inquiries can be directed to the corresponding author.

## Ethics Statement

The studies involving human participants were reviewed and approved by University at Buffalo/Oishei Children’s Hospital of Buffalo, and Nationwide Children’s Hospital. Written informed consent to participate in this study was provided by the participants’ legal guardian/next of kin.

## Author Contributions**

All authors contributed to the study conception and design. Material preparation, data collection and analysis were performed by all authors. The first draft of the manuscript was written by LeM and all authors commented on previous versions of the manuscript. All authors contributed to the article and approved the submitted version.

## Funding

Study was funded by the fellow’s division funding at University at Buffalo, NY and the Research Institute, Nationwide Children’s Hospital, Columbus, OH, USA.

## Conflict of Interest

Author LuM has received research grants from Novo Nordisk, Astrazeneca, Sanofi Aventis, and from Juvenile Diabetes Research Foundation.

The remaining authors declare that the research was conducted in the absence of any commercial or financial relationships that could be construed as a potential conflict of interest.
